# Association of platelet-rich plasma combined with dorsal root ganglion-targeted pulsed radiofrequency with outcomes in subacute thoracic herpes zoster neuralgia: a retrospective cohort study

**DOI:** 10.3389/fneur.2026.1833755

**Published:** 2026-09-28

**Authors:** Xiaolong Ye, Xin Hu, Zenghua Zhou, Shengrong Xu, Ruilin He, Zongbin Jiang

**Affiliations:** Department of Pain Medicine, The Second Affiliated Hospital of Guangxi Medical University, Nanning, Guangxi, China

**Keywords:** dorsal root ganglion, persistent clinically meaningful pain, platelet-rich plasma, pulsed radiofrequency, subacute herpes zoster neuralgia, zoster-associated pain

## Abstract

**Background:**

Subacute herpes zoster neuralgia is a clinically important transitional stage before pain becomes fully established as persistent postherpetic neuralgia.

**Methods:**

In this single-center retrospective cohort study, patients with subacute thoracic herpes zoster neuralgia treated between January 2024 and December 2025 were identified from routine clinical records. Patients were classified according to the treatment received as PRP + PRF or PRF alone. The primary outcome was the Numerical Rating Scale (NRS) score at 3 months after treatment. Repeated NRS and Pittsburgh Sleep Quality Index (PSQI) measurements were analyzed longitudinally. Secondary outcomes included pregabalin requirement and persistent clinically meaningful zoster-related pain, defined as NRS > 3 at the 3-month post-treatment follow-up.

**Results:**

Seventy patients were analyzed, including 36 in the PRP + PRF group and 34 in the PRF group. At 3 months, the observed mean NRS score was lower in the PRP + PRF group than in the PRF group (1.39 ± 1.44 vs. 2.56 ± 1.02). In the prespecified ANCOVA adjusted for baseline NRS, PRP + PRF was associated with a lower 3-month NRS score than PRF alone (adjusted mean difference, −1.13; 95% confidence interval [CI], −1.71 to −0.54; *p* < 0.001). The association remained directionally consistent in additional multivariable and change-score sensitivity analyses. Significant group-by-time interactions were observed for NRS and PSQI, with lower NRS and PSQI scores in the PRP + PRF group from 1 week through 3 months. Pregabalin requirement at 3 months was lower in the PRP + PRF group (*p* = 0.001). Persistent clinically meaningful zoster-related pain at 3 months occurred in 4 of 36 patients (11.1%) in the PRP + PRF group and 7 of 34 patients (20.6%) in the PRF group (*p* = 0.336).

**Conclusion:**

In this retrospective cohort, PRP combined with DRG-targeted PRF was associated with faster early improvement in pain and sleep and with lower post-treatment pregabalin use than PRF alone. Further prospective studies are needed to confirm these findings and determine whether this approach influences progression to persistent pain and PHN.

## 1 Introduction

Herpes zoster results from reactivation of latent varicella-zoster virus in sensory ganglia, including the dorsal root ganglia, and is frequently accompanied by neuropathic pain distributed along the involved dermatome (1–3). In some patients, pain persists beyond the acute eruptive stage and progresses to postherpetic neuralgia (PHN), a complication whose frequency and duration increase with age (4–6). In addition to pain itself, zoster-associated neuralgia substantially impairs sleep, quality of life, and daily functioning (7–9).

The subacute stage is clinically important because it represents a period during which zoster-associated pain is no longer limited to the acute cutaneous eruption, yet may not have become fully established as persistent chronic pain. Mechanistically, ongoing virus-related injury in primary sensory neurons, persistent ectopic afferent activity, and evolving central sensitization may all contribute to pain persistence during this stage (10, 11). From a clinical perspective, treatment timing appears to matter: delayed treatment has been associated with a higher risk of PHN, and earlier interventional treatment has been associated with better outcomes in zoster-related neuralgia (12, 13).

Current treatment remains challenging. Although pharmacologic therapy is widely used, analgesia is often incomplete, and treatment may be limited by adverse effects, dose titration, and the complexity of managing older patients with comorbidity and polypharmacy (14–17). Interventional approaches targeting the dorsal root ganglion (DRG) have therefore attracted attention. Pulsed radiofrequency (PRF) applied to the DRG has shown benefit in acute and subacute zoster-related pain, and earlier intervention may be associated with better outcomes than delayed treatment after pain has become more established (13, 18–20).

PRF, however, is primarily a neuromodulatory intervention and may not fully address inflammatory and reparative components of zoster-associated neural injury. Platelet-rich plasma (PRP) is an autologous blood-derived product enriched in platelets and bioactive mediators. Experimental and translational studies suggest that PRP may support peripheral nerve repair and modulate the local inflammation (21–24). Clinical interest in PRP for zoster-associated pain has also increased. Prior clinical studies have reported favorable findings with PRP in herpes zoster neuralgia (25), and intralesional PRP has shown preliminary benefit in PHN (26). Recent clinical and review-level evidence has suggested that PRP-based approaches, including PRP combined with PRF, may improve pain, sleep, and medication use in zoster-associated pain, although the current evidence base remains limited and heterogeneous (27–30).

Accordingly, this retrospective cohort study evaluated the association of PRP combined with DRG-targeted PRF with clinical outcomes in patients with subacute thoracic herpes zoster neuralgia and compared outcomes with those of PRF alone. We hypothesized that, during this subacute window, the addition of PRP would be associated with greater early improvement in pain and sleep and reduced reliance on adjunctive pregabalin.

## 2 Methods

### 2.1 Study design

This was a single-center, unmatched, non-randomized retrospective cohort study conducted in the Department of Pain Medicine, the Second Affiliated Hospital of Guangxi Medical University, between January 2024 and December 2025. The study was approved by the Ethics Committee of the Second Affiliated Hospital of Guangxi Medical University (2026-KY[023]). Owing to the retrospective nature of the study, the requirement for informed consent was waived. The study was reported in accordance with the Strengthening the Reporting of Observational Studies in Epidemiology (STROBE) framework.

### 2.2 Patient selection

Patients with subacute herpes zoster neuralgia who underwent DRG-targeted PRF with or without PRP during the study period were screened through review of the electronic medical records. Patients with thoracic involvement who met the eligibility criteria were included in the final analytic cohort. Subacute herpes zoster neuralgia was defined as persistent zoster-related pain 30 to 90 days after rash onset.

Inclusion criteria were: (1) a diagnosis of subacute thoracic herpes zoster neuralgia; (2) unilateral pain distributed along one or more thoracic dermatomes; and (3) treatment with DRG-targeted PRF performed under fluoroscopic guidance using a digital subtraction angiography (DSA) system, either alone or combined with PRP.

*Exclusion criteria were:* (1) local infection at the puncture site or active systemic infection; (2) coagulation dysfunction, significant hematological abnormalities, or any other condition that would preclude safe autologous blood collection and PRP preparation; (3) severe psychiatric disorders or cognitive impairment that would interfere with treatment compliance or outcome assessment; (4) previous interventional treatment for the same episode of thoracic herpes zoster neuralgia prior to the index procedure; and (5) missing key baseline demographic, clinical, or laboratory data, or missing primary/secondary outcome data at any prespecified assessment time point.

### 2.3 Treatment allocation and groups

Patients were assigned to two groups according to the treatment they actually received at the index procedure: a combined PRP and PRF group, and a PRF monotherapy group. Treatment allocation was non-randomized, consistent with the retrospective observational study design. During the study period, the decision to administer adjunctive PRP was made via shared clinical decision-making between the treating physician and the patient in a real-world practice setting, without adherence to a formal randomization protocol. Potential confounders of treatment selection included patient preference, physician discretion, perceived symptom burden, and patient willingness to receive an additional autologous biologic injection. Owing to the retrospective nature of the study and the risk of unmeasured confounding, our findings should be interpreted as observational associations rather than causal inferences. To mitigate this limitation, we performed additional covariate-adjusted sensitivity analyses to confirm the robustness of the primary results.

### 2.4 Interventions

#### 2.4.1 DSA-guided thoracic DRG-targeted procedure

All procedures were performed by experienced pain physicians under fluoroscopic guidance using a digital subtraction angiography system and under sterile conditions (Figure 1). Patients were placed in the prone position. The target segment was determined according to the dermatomal distribution of pain and the corresponding vertebral level. After routine skin disinfection and sterile draping, a disposable puncture needle (CZ01-L; Sichuan Jinjiang Electronic Technology Co., Ltd., China) was advanced toward the DRG region adjacent to the involved thoracic intervertebral foramen under anteroposterior and lateral fluoroscopic views. Needle position was adjusted until the tip reached the intended target region. Contrast medium was not routinely administered prior to PRP injection in our clinical protocol to prevent potential interference with PRP bioactivity; final positioning was based on fluoroscopic views together with sensory and motor stimulation. Before any injection, negative aspiration was performed to confirm the absence of blood or cerebrospinal fluid.

**Figure 1:**
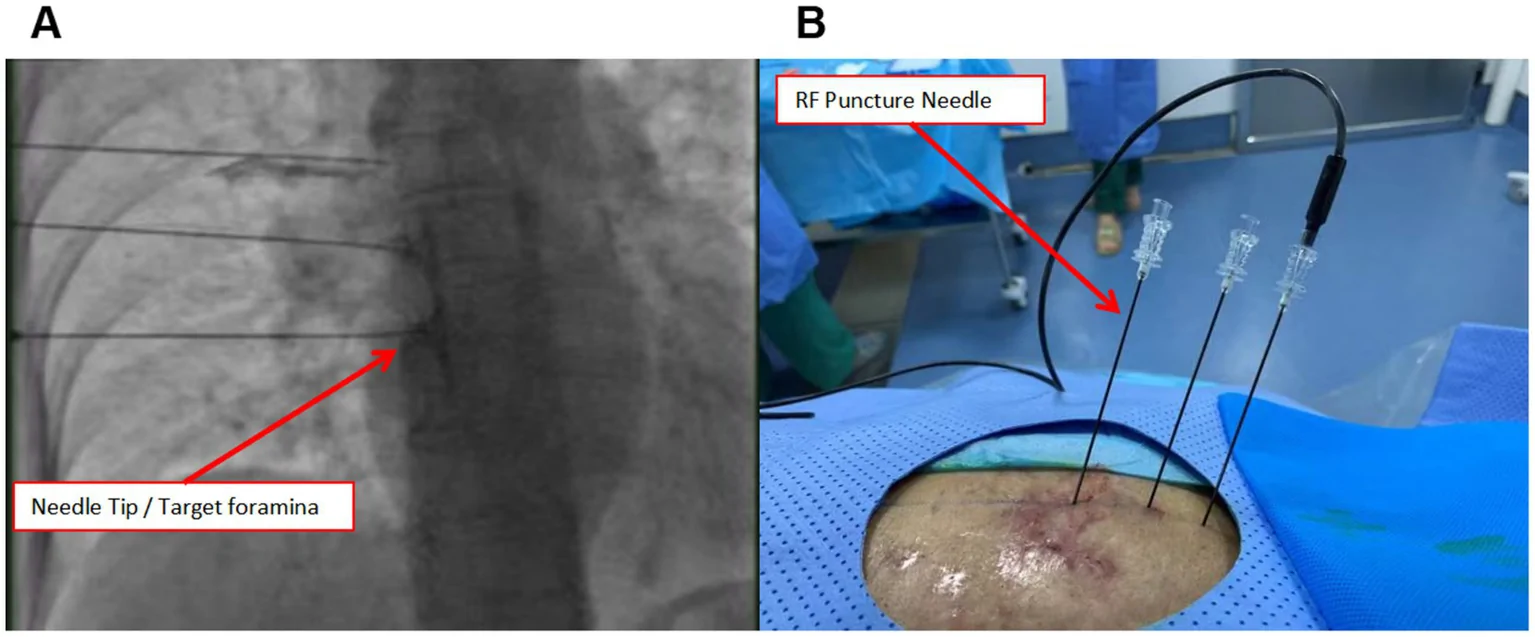
Representative procedural images of dorsal root ganglion (DRG)-targeted pulsed radiofrequency. **(A)** Representative anteroposterior fluoroscopic image showing needle placement toward the target thoracic intervertebral foramina. The labels indicate the approximate needle tip/target foramen region on the representative de-identified image. **(B)** Representative intraoperative photograph showing percutaneous radiofrequency trocars in place before PRF. Final target confirmation was based on combined anteroposterior/lateral fluoroscopy and sensory/motor stimulation, not surface appearance alone. PRF, pulsed radiofrequency.

#### 2.4.2 PRF procedure

After satisfactory needle placement, the radiofrequency electrode was connected to a radiofrequency generator (VATION-50 N; Sichuan Jinjiang Electronic Technology Co., Ltd., China). The final target position was confirmed by sensory and motor stimulation, with concordant segmental sensation and the absence of unacceptable motor responses. PRF was applied at a frequency of 2 Hz, a pulse width of 20 ms, and a temperature of 42 °C for 300 s; this cycle was repeated twice.

#### 2.4.3 PRP preparation and administration

In the PRP + PRF group, autologous PRP was prepared immediately before injection using a standardized commercial preparation protocol. The main equipment used was a platelet-rich plasma preparation device (CLZD-I) and its dedicated centrifuge (CLZD-LXJ3-X2) manufactured by Beijing Chunli Zhengda Medical Devices Co., Ltd., Beijing, China. According to the number of target segments, 30 mL of peripheral venous blood was collected via a single venipuncture, and 3 mL of 3.2% sodium citrate anticoagulant was pre-drawn before blood collection. Approximately 2–4 mL of PRP was prepared according to the manufacturer’s protocol and the number of target segments. After completion of the PRF procedure, PRP was injected into the target DRG region through the same radiofrequency puncture cannula at a dose of 1 mL per target segment. After the injection, patients were observed for approximately 30 min.

PRP preparation for all enrolled patients followed the above unified standard protocol. Because the eligibility criteria excluded patients with coagulation dysfunction, abnormal hematological tests, or inability to undergo puncture or PRP preparation, major contraindications to autologous blood collection or PRP preparation were excluded before analysis. In this study, routine measurements of platelet concentration and its fold increase relative to baseline whole blood, leukocyte-rich/leukocyte-poor classification, erythrocyte contamination level, and activation status were not performed under a formal quality control system. No exogenous activators were used to activate PRP.

#### 2.4.4 PRF-alone group

Patients in the PRF group underwent the same DSA-guided thoracic DRG-targeted PRF procedure but did not receive PRP injection.

#### 2.4.5 Safety assessment

Procedure-related adverse events documented in the medical records during the procedure, the immediate post-procedure observation period, and follow-up visits were reviewed. Events of interest included pneumothorax, bleeding, infection, fever, local hematoma, transient pain exacerbation, numbness or new sensory symptoms, vasovagal reaction, dizziness, new neurologic deficits, readmission, and other unexpected events. Because adverse events were identified retrospectively from routine records, minor transient or delayed events may have been underreported.

### 2.5 Concomitant treatment

Concomitant analgesic treatment consisted primarily of pregabalin, which was prescribed and adjusted according to pain severity, treatment response, and tolerability at the discretion of the treating physicians. There was no prespecified uniform pregabalin titration or tapering protocol. The daily pregabalin dose was recorded at baseline and at the 3-month follow-up. Accordingly, 3-month pregabalin dose was interpreted as a post-treatment medication-use outcome and possible mediator of symptom improvement rather than as an independent causal efficacy endpoint. Other concomitant treatments, including antiviral therapy, corticosteroids, nonsteroidal anti-inflammatory drugs, opioids, topical analgesics, and rescue medications, were not consistently captured in the structured dataset and could not be adjusted for quantitatively.

### 2.6 Outcomes and follow-up

Clinical assessments were performed at baseline before treatment and at 1 week, 1 month, 3 months, and 6 months after treatment. The primary outcome was pain intensity at 3 months after treatment, assessed using the Numerical Rating Scale (NRS).

Secondary outcomes included: (1) longitudinal NRS scores across all follow-up time points; (2) longitudinal Pittsburgh Sleep Quality Index (PSQI) scores; (3) pregabalin requirement at 3 months after treatment; and (4) the proportion of patients with persistent clinically meaningful zoster-related pain at the 3-month post-treatment follow-up. This endpoint was defined operationally as NRS > 3 at the 3-month post-treatment follow-up. Because patients were treated 30 to 90 days after rash onset, this visit occurred beyond 90 days after rash onset in all included patients, but the exact post-rash interval varied. Therefore, this endpoint was not presented as standard PHN incidence.

### 2.7 Statistical analysis

All statistical analyses were performed using R version 4.4.1 (R Foundation for Statistical Computing, Vienna, Austria). All tests were two-sided, and p < 0.05 was considered statistically significant.

For baseline characteristics, approximately normally distributed continuous variables were summarized as mean ± standard deviation and compared between groups using Welch’s two-sample *t* test. Non-normally distributed continuous variables were summarized as median (interquartile range) and compared using the Wilcoxon rank-sum test. Categorical variables were summarized as n (%) and compared using Fisher’s exact test.

Missing data were handled by complete-case analysis. Complete key baseline variables and prespecified follow-up outcomes were required for inclusion in the analytic cohort; therefore, no missing values remained for the primary outcome or longitudinal NRS and PSQI measurements in the final dataset, and no statistical imputation was performed. Patients excluded because of incomplete clinical or follow-up data are shown in the study flow diagram.

The primary outcome was formally analyzed using analysis of covariance (ANCOVA), with 3-month NRS score as the dependent variable, treatment group as the main independent variable, and baseline NRS score as a covariate. The prespecified model was T3NRS ~ Group + T0NRS. Adjusted between-group mean differences, 95% confidence intervals (CIs), and *p* values were reported.

NRS and PSQI scores were analyzed as continuous outcomes to estimate between-group mean differences, consistent with the prespecified analytic approach. The primary ANCOVA result was interpreted together with observed longitudinal trajectories, the change-score sensitivity analysis, and the binary endpoint of persistent clinically meaningful zoster-related pain.

To address potential confounding, additional sensitivity analyses were performed using clinically interpretable models rather than highly parameterized causal models. These included ANCOVA models additionally adjusted for age, sex, disease duration, baseline PSQI score, baseline pregabalin dose, rash extent, hyperalgesia, diabetes mellitus, and hypertension, as well as a change-score sensitivity analysis from baseline to 3 months. Because the sample size was modest, these models were interpreted as sensitivity analyses rather than definitive causal adjustment.

Repeated measurements of NRS and PSQI were analyzed using linear mixed-effects models. Fixed effects included group, time, and the group-by-time interaction, and a subject-specific random intercept was included to account for within-patient correlation. Type III tests were used for fixed effects, with denominator degrees of freedom estimated by the Satterthwaite method. Between-group comparisons at each time point were derived from estimated marginal means obtained from the final mixed-effects models, using the Kenward-Roger method for degrees-of-freedom estimation. These time-specific contrasts were treated as supportive exploratory analyses. Unadjusted *p* values were reported together with Bonferroni-adjusted *p* values as a sensitivity analysis for multiple time-specific comparisons.

Pregabalin dose at 3 months was summarized as median (interquartile range) and compared using the Wilcoxon rank-sum test. The proportion of patients with persistent clinically meaningful zoster-related pain at 3 months was compared using Fisher’s exact test.

## 3 Results

### 3.1 Study cohort and baseline characteristics

A total of 96 patients with subacute herpes zoster neuralgia who underwent DRG-targeted PRF therapy with or without PRP injection between January 1, 2024, and December 31, 2025, were initially screened. Thirteen patients with non-thoracic involvement were excluded, leaving 83 eligible patients. Patients were then grouped according to the actual treatment they received: 44 received combined PRP and PRF therapy, and 39 received PRF. Eight patients in the combination group were further excluded due to incomplete clinical or follow-up data or prior interventional treatment for the same episode of neuralgia, and 5 patients in the PRF group were excluded for the same reasons (Figure 2).

**Figure 2:**
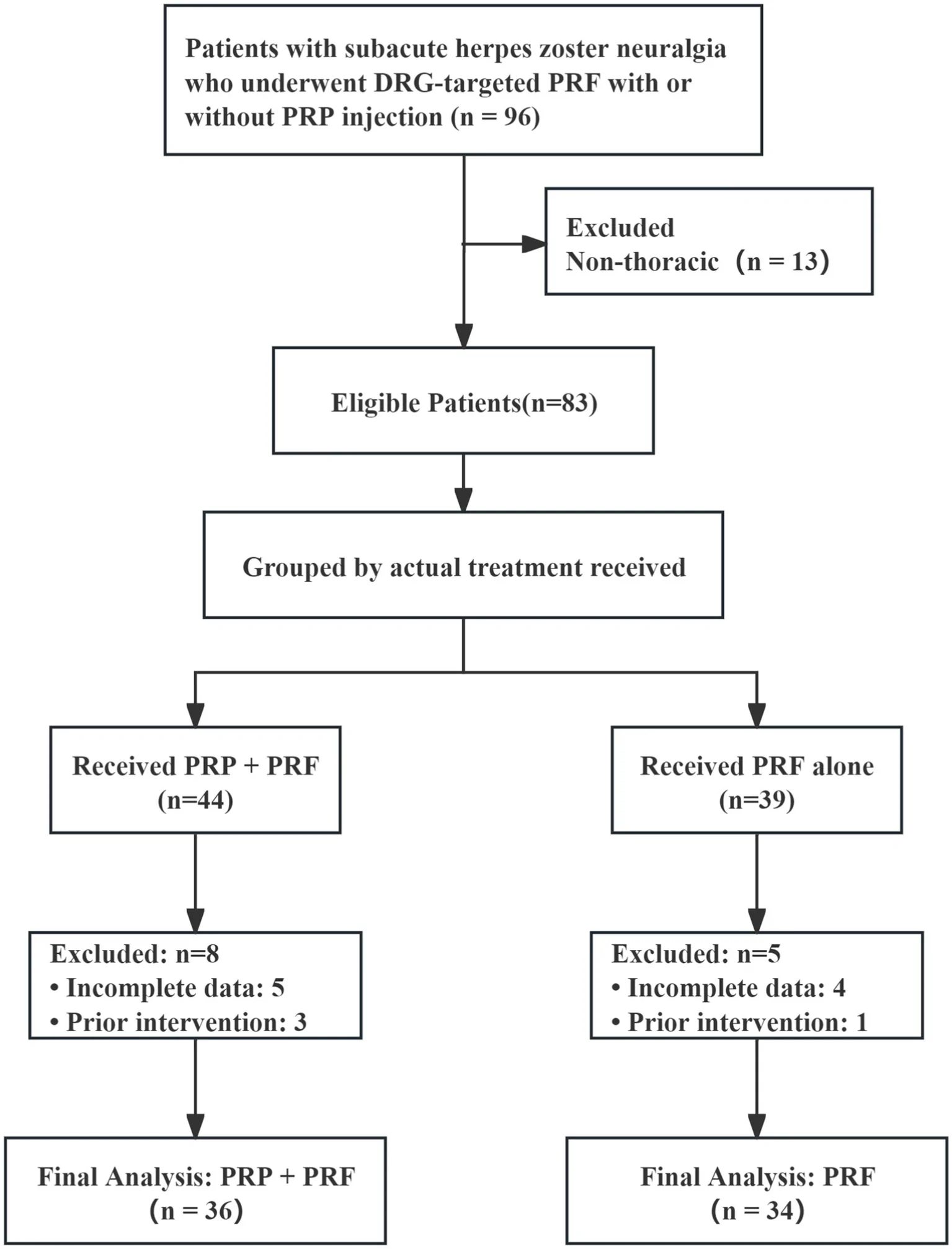
Flow chart of the retrospective cohort selection process. This flow chart summarizes the identification, exclusion, treatment grouping, and final inclusion of patients in the retrospective cohort. Among 96 patients screened during the study period, 13 were excluded because of non-thoracic involvement. The remaining 83 eligible patients were classified according to the treatment actually received, with 44 patients in the PRP + PRF group and 39 patients in the PRF-alone group. After excluding patients with incomplete clinical or follow-up data or prior interventional treatment for the same episode of neuralgia, 36 patients in the PRP + PRF group and 34 patients in the PRF group were included in the final analysis. PRF, pulsed radiofrequency; PRP, platelet-rich plasma.

Ultimately, 70 patients with subacute thoracic herpes zoster neuralgia were included in the final analysis, with 36 in the PRP + PRF group and 34 in the PRF group. Measured baseline demographic and clinical characteristics were broadly comparable between the two groups (Table 1). No statistically significant between-group differences were observed for the listed baseline variables (*p* > 0.05), although baseline comparability based on measured variables should not be interpreted as full exchangeability in a non-randomized cohort.

**Table 1:** Baseline demographic and clinical characteristics of the study population.

Variable	PRP + PRF (*n* = 36)	PRF (*n* = 34)	*p* value
Age, years	64.11 ± 10.33	65.56 ± 9.20	0.537
Sex, *n* (%)			0.808
Male	14 (38.9)	12 (35.3)	
Female	22 (61.1)	22 (64.7)	
BMI, kg/m^2^	22.66 ± 3.23	23.50 ± 3.49	0.302
Disease duration, days	38.00 (34.75–41.25)	38.50 (35.00–41.00)	0.465
Marital status, *n* (%)			0.43
Single	5 (13.9)	2 (5.9)	
Married	31 (86.1)	32 (94.1)	
Pain side, *n* (%)			0.161
Left	20 (55.6)	13 (38.2)	
Right	16 (44.4)	21 (61.8)	
Prodromal symptoms, *n* (%)			0.626
No	13 (36.1%)	15 (44.1%)	
Yes	23 (63.9%)	19 (55.9%)	
Rash extent, *n* (%)			0.475
Small area	9 (25.0%)	13 (38.2%)	
Medium area	12 (33.3%)	8 (23.5%)	
Large area	15 (41.7%)	13 (38.2%)	
Pain character, *n* (%)			0.211
Burning	13 (36.1)	15 (44.1)	
Pricking	14 (38.9)	16 (47.1)	
Electric shock	9 (25.0)	3 (8.8)	
Hyperalgesia, *n* (%)			0.247
No	10 (27.8)	5 (14.7)	
Yes	26 (72.2)	29 (85.3)	
Smoking status, *n* (%)			0.551
Never	26 (72.2)	28 (82.4)	
Current	5 (13.9)	4 (11.8)	
Former	5 (13.9)	2 (5.9)	
Alcohol use, *n* (%)			0.138
Never	22 (61.1)	20 (58.8)	
Current	7 (19.4)	12 (35.3)	
Former	7 (19.4)	2 (5.9)	
Hypertension, *n* (%)			0.315
No	22 (61.1%)	25 (73.5%)	
Yes	14 (38.9%)	9 (26.5%)	
Diabetes mellitus, *n* (%)			0.261
No	30 (83.3)	32 (94.1)	
Yes	6 (16.7)	2 (5.9)	
Tumor history, *n* (%)			0.493
No	34 (94.4)	34 (100.0)	
Yes	2 (5.6)	0 (0.0)	
Baseline NRS score	6.92 ± 0.91	7.09 ± 1.48	0.565
Baseline PSQI score	12.61 ± 2.25	12.97 ± 2.48	0.528
Baseline pregabalin dose, mg/day	300.00 (150.00–300.00)	150.00 (150.00–600.00)	0.307

Data are presented as mean ± standard deviation, median (interquartile range), or *n* (%), as appropriate. *p* values were calculated using Welch’s two-sample *t* test, Wilcoxon rank-sum test, or Fisher’s exact test. BMI, body mass index; NRS, Numerical Rating Scale; PSQI, Pittsburgh Sleep Quality Index; PRF, pulsed radiofrequency; PRP, platelet-rich plasma.

### 3.2 Primary outcome: pain intensity at 3 months

At 3 months after treatment, the observed mean NRS score was lower in the PRP + PRF group than in the PRF group (1.39 ± 1.44 vs. 2.56 ± 1.02; Table 2). In the prespecified ANCOVA adjusted for baseline NRS score, treatment group remained significantly associated with pain intensity at 3 months. Compared with PRF alone, PRP + PRF was associated with a lower 3-month NRS score, with an adjusted mean difference of −1.13 (95% CI, −1.71 to −0.54; *p* < 0.001). A supportive repeated-measures analysis using the linear mixed-effects model yielded a consistent estimate at the same time point (mean difference, −1.17; 95% CI, −1.77 to −0.57; *p* < 0.001) (Table 2; Figure 3A).

**Table 2:** Observed longitudinal outcomes and model-based between-group contrasts.

Outcome	Time point	PRP + PRF (*n* = 36)	PRF (n = 34)	Mean difference (95% CI)*	*p* value (unadjusted)	*p* value (Bonferroni)
NRS	Baseline	6.92 ± 0.91	7.09 ± 1.48	−0.17 (−0.77 to 0.43)	0.573	1.000
NRS	1 week	2.94 ± 1.12	4.06 ± 1.69	−1.11 (−1.71 to −0.51)	<0.001	0.002
NRS	1 month	2.19 ± 1.21	3.15 ± 1.13	−0.95 (−1.55 to −0.35)	0.002	0.010
NRS	3 months	1.39 ± 1.44	2.56 ± 1.02	−1.17 (−1.77 to −0.57)	<0.001	<0.001
NRS	6 months	1.08 ± 1.11	1.68 ± 1.43	−0.59 (−1.19 to 0.01)	0.053	0.263
PSQI	Baseline	12.61 ± 2.25	12.97 ± 2.48	−0.36 (−1.69 to 0.97)	0.595	1.000
PSQI	1 week	8.31 ± 2.70	11.12 ± 4.03	−2.81 (−4.14 to −1.48)	<0.001	<0.001
PSQI	1 month	5.81 ± 4.05	8.26 ± 2.59	−2.46 (−3.79 to −1.13)	<0.001	0.002
PSQI	3 months	3.94 ± 2.78	6.91 ± 2.33	−2.97 (−4.30 to −1.64)	<0.001	<0.001
PSQI	6 months	2.92 ± 1.68	4.24 ± 2.44	−1.32 (−2.65 to 0.01)	0.052	0.261

Continuous longitudinal outcomes are presented as observed mean ± standard deviation. Between-group mean differences are shown as PRP + PRF minus PRF and were estimated from the linear mixed-effects model. Unadjusted *p* values correspond to exploratory post hoc contrasts of estimated marginal means. Bonferroni-adjusted *p* values were calculated as a sensitivity analysis for the five time-specific comparisons within each outcome. In the prespecified ANCOVA adjusted for baseline NRS score, the adjusted mean difference in NRS score at 3 months was −1.13 (95% CI, −1.71 to −0.54; *p* < 0.001). CI, confidence interval; NRS, Numerical Rating Scale; PSQI, Pittsburgh Sleep Quality Index.

**Figure 3:**
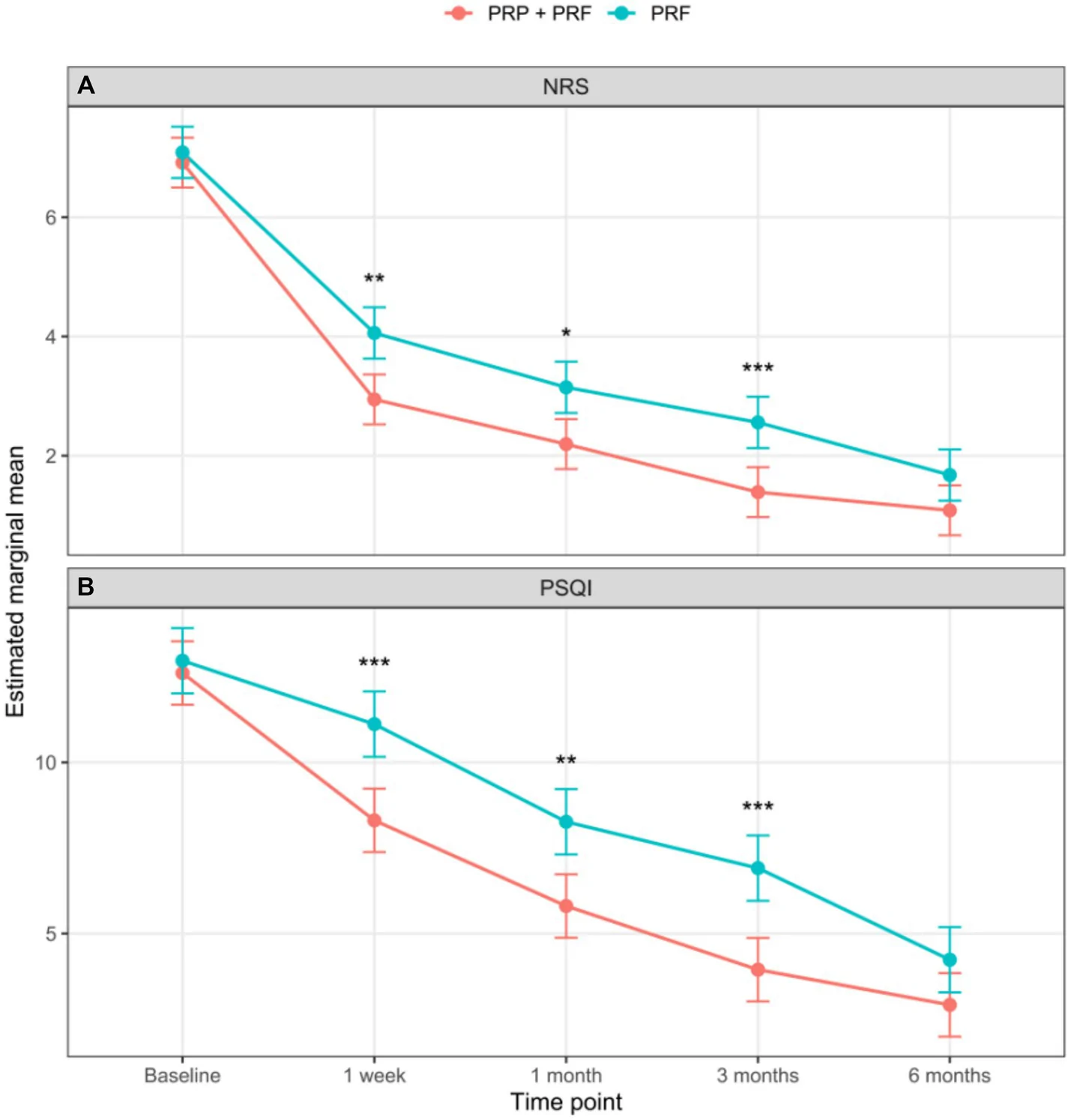
Trajectories of pain intensity and sleep quality from baseline to 6 months after treatment. **(A)** Numerical Rating Scale (NRS) scores. **(B)** Pittsburgh Sleep Quality Index (PSQI) scores. Points represent estimated marginal means derived from the linear mixed-effects model, and error bars indicate 95% confidence intervals. Asterisks indicate Bonferroni-adjusted time-specific between-group comparisons (*p < 0.05; **p < 0.01; ***p < 0.001). No statistically significant between-group differences were detected at baseline or at 6 months. NRS, Numerical Rating Scale; PRF, pulsed radiofrequency; PRP, platelet-rich plasma; PSQI, Pittsburgh Sleep Quality Index.

### 3.3 Sensitivity analyses for the primary outcome

The primary association remained directionally consistent across additional sensitivity analyses (Table 3). In models additionally adjusted for age, sex, disease duration, baseline PSQI score, baseline pregabalin dose, rash extent, hyperalgesia, diabetes mellitus, and hypertension, the estimated between-group difference in 3-month NRS score ranged from −0.99 to −1.01 and remained statistically significant. The change-score sensitivity analysis also favored PRP + PRF, with a greater NRS reduction from baseline to 3 months (between-group difference, 1.13; 95% CI, 0.54 to 1.71; *p* < 0.001).

**Table 3:** Sensitivity analyses for the primary 3-month NRS outcome.

Model	Effect estimate	95% CI	*p* value
Primary ANCOVA: group + baseline NRS	−1.13	−1.71 to −0.54	<0.001
Adjusted model 1: + age, sex, disease duration	−0.99	−1.55 to −0.43	<0.001
Adjusted model 2: + baseline PSQI and baseline pregabalin	−1.01	−1.58 to −0.45	<0.001
Adjusted model 3: + selected clinical covariates	−1.03	−1.66 to −0.40	0.002
Change-score sensitivity: NRS reduction from baseline	1.13	0.54 to 1.71	<0.001

For models with 3-month NRS score as the dependent variable, negative values favor PRP + PRF. For the change-score model, positive values indicate greater NRS reduction in the PRP + PRF group. The selected clinical covariate model adjusted for age, sex, disease duration, baseline NRS, baseline PSQI, baseline pregabalin dose, rash extent, hyperalgesia, diabetes mellitus, and hypertension. Number of treated thoracic segments was not available in the structured dataset. ANCOVA, analysis of covariance; CI, confidence interval; NRS, Numerical Rating Scale; PSQI, Pittsburgh Sleep Quality Index.

### 3.4 Longitudinal changes in pain intensity and sleep quality

NRS scores decreased over time in both groups (Figure 3A). In the linear mixed-effects model, significant effects were observed for group (*F*[1, 68] = 12.75, *p* < 0.001), time (*F*[4, 272] = 370.32, *p* < 0.001), and the group-by-time interaction (*F*[4, 272] = 3.29, *p* = 0.012) (Table 4). Time-specific contrasts showed lower NRS scores in the PRP + PRF group at 1 week, 1 month, and 3 months, and these differences remained significant after Bonferroni adjustment. No statistically significant between-group difference was detected at baseline or at 6 months (Table 2).

**Table 4:** Type III tests from linear mixed-effects models for repeated NRS and PSQI measurements.

Outcome	Fixed effect	F statistic	*p* value
NRS	Group	*F*(1, 68) = 12.75	<0.001
NRS	Time	*F*(4, 272) = 370.32	<0.001
NRS	Group: Time	*F*(4, 272) = 3.29	0.012
PSQI	Group	*F*(1, 68) = 16.01	<0.001
PSQI	Time	*F*(4, 272) = 200.33	<0.001
PSQI	Group: Time	*F*(4, 272) = 4.73	0.001

Linear mixed-effects models included fixed effects for group, time, and the group-by-time interaction, with a subject-specific random intercept. Degrees of freedom and F statistics were estimated using Satterthwaite’s approximation. NRS, Numerical Rating Scale; PSQI, Pittsburgh Sleep Quality Index.

PSQI scores also decreased over time in both groups (Figure 3B). In the linear mixed-effects model, significant effects were observed for group (*F*[1, 68] = 16.01, *p* < 0.001), time (*F*[4, 272] = 200.33, *p* < 0.001), and the group-by-time interaction (*F*[4, 272] = 4.73, *p* = 0.001) (Table 4). Time-specific contrasts showed lower PSQI scores in the PRP + PRF group at 1 week, 1 month, and 3 months, and these differences remained significant after Bonferroni adjustment. No statistically significant between-group difference was detected at baseline or at 6 months (Table 2).

### 3.5 Pregabalin requirement and persistent clinically meaningful pain at 3 months

At 3 months after treatment, pregabalin requirement was lower in the PRP + PRF group than in the PRF group, with median doses of 0.00 (IQR, 0.00–150.00) mg/day and 150.00 (IQR, 150.00–300.00) mg/day, respectively (*p* = 0.001). Because pregabalin dose was adjusted according to clinical response and tolerability rather than a standardized tapering protocol, this finding was interpreted as a post-treatment medication-use marker that may partly reflect pain improvement.

At the 3-month post-treatment follow-up, 4 of 36 patients (11.1%) in the PRP + PRF group and 7 of 34 patients (20.6%) in the PRF group had persistent clinically meaningful zoster-related pain, corresponding to an absolute between-group difference of −9.5 percentage points. This between-group difference was not statistically significant (Fisher’s exact test, *p* = 0.336) (Figure 4).

**Figure 4:**
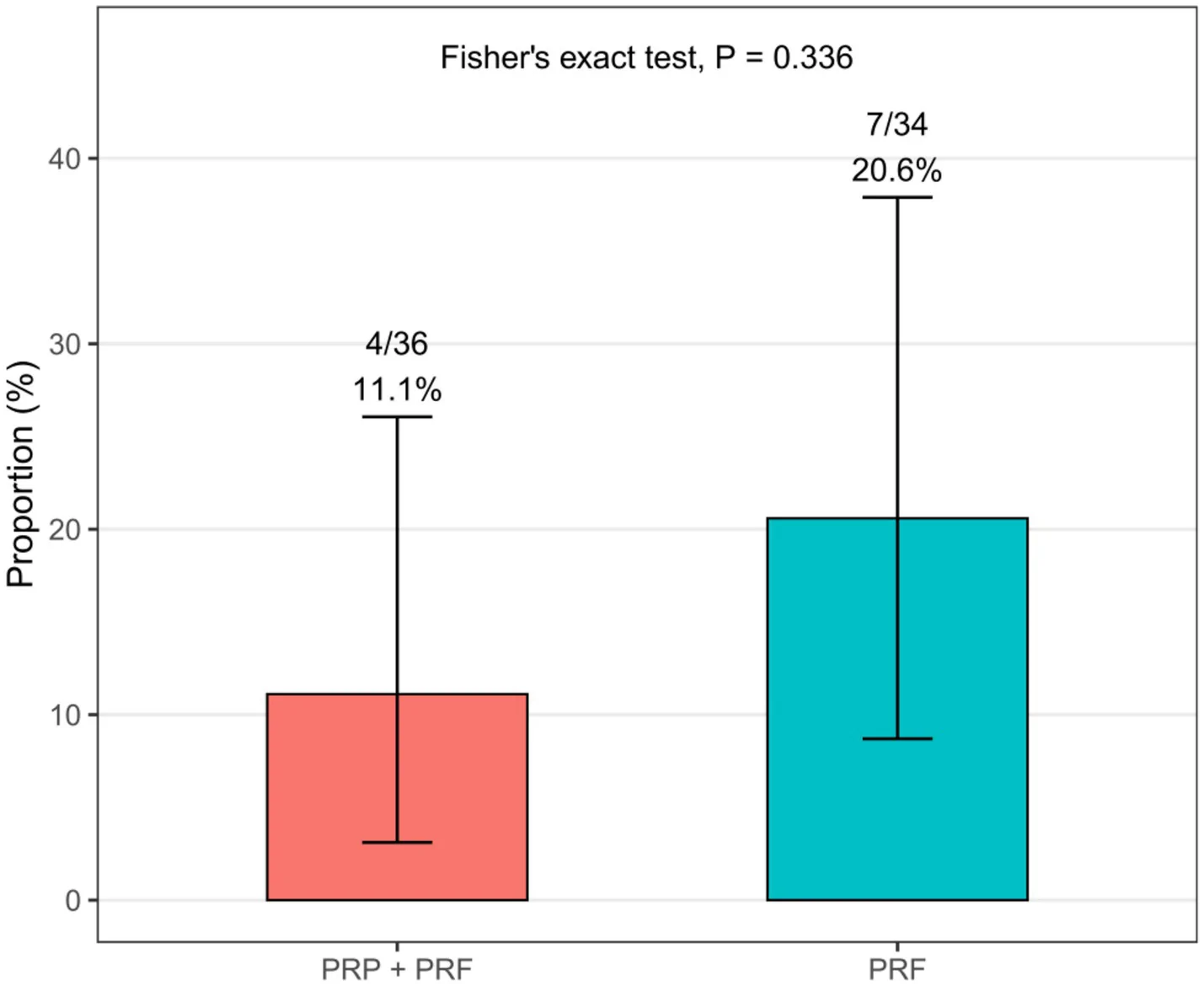
Proportion of patients with persistent clinically meaningful zoster-related pain at the 3-month post-treatment follow-up. Bars represent the percentage of patients meeting the operational endpoint of persistent clinically meaningful zoster-related pain, defined as NRS > 3 at 3 months after treatment. Error bars indicate 95% exact binomial confidence intervals. The between-group comparison was performed using Fisher’s exact test (*p* = 0.336). This endpoint was exploratory and should not be interpreted as standardized postherpetic neuralgia incidence. NRS, Numerical Rating Scale; PHN, postherpetic neuralgia; PRF, pulsed radiofrequency; PRP, platelet-rich plasma.

### 3.6 Adverse events

No procedure-related serious adverse events were documented in either group. Specifically, the available records did not document pneumothorax, clinically significant bleeding, infection, fever, local hematoma requiring treatment, transient pain exacerbation requiring additional intervention, numbness or new sensory symptoms, dizziness requiring treatment, vasovagal reaction, readmission, or new neurologic deficit. Because this was a retrospective review, minor transient symptoms or delayed events not recorded in routine notes may have been missed.

## 4 Discussion

In this single-center non-randomized retrospective cohort study of patients with subacute thoracic herpes zoster neuralgia, adding autologous PRP to DRG-targeted PRF was associated with lower 3-month NRS scores, greater early improvement in PSQI scores, and lower pregabalin requirement at 3 months. The association with the 3-month NRS outcome remained directionally consistent in multivariable covariate-adjusted and change-from-baseline sensitivity analyses. These findings should nevertheless be interpreted as associative rather than causal because treatment allocation was not randomized and several clinically relevant variables were not fully standardized or recorded.

The timing of intervention is important. The subacute phase of zoster-associated pain is not merely a residual period after rash eruption. It is a transitional stage during which pain may still be driven by ongoing dysfunction of primary sensory neurons, persistent abnormal peripheral input, and progressive sensitization within the nociceptive system (10, 11). Because varicella-zoster virus is latent in sensory ganglia, including the DRG, ganglion-level pathology is central to the pathogenesis of zoster-associated pain and PHN (1, 2, 31). Prior clinical studies of DRG-targeted PRF have suggested that earlier intervention may be more favorable before pain becomes entrenched as refractory PHN (13, 18–20). In this context, the significant group-by-time interactions observed in our study suggest that PRP + PRF may be associated with faster early recovery from 1 week through 3 months, rather than a durable separation that persisted unequivocally through 6 months.

The observed early advantage of combination therapy is biologically plausible, although not mechanistically proven by this study. PRF is generally regarded as a neuromodulatory treatment directed at the DRG, where it may reduce abnormal afferent discharge and attenuate pain amplification. Experimental data also suggest that PRF can reduce mechanical hypersensitivity and may attenuate neuroinflammatory signaling (32). PRP provides a biologically different intervention, as platelet-derived mediators have been implicated in tissue repair, peripheral nerve regeneration, and modulation of local inflammation (21–24). This complementary rationale is relevant in subacute herpes zoster neuralgia, in which neural injury and maladaptive neuroinflammation may coexist. However, because platelet concentration, leukocyte content, and other PRP characteristics were not measured, the present data cannot determine which PRP components were responsible for the observed association.

The sleep findings are clinically relevant. Herpes zoster-related pain can substantially disrupt sleep and quality of life, and sleep shortage has been associated with subsequent PHN development in epidemiologic data (7–9, 33). In our study, PSQI scores decreased more rapidly in the combination group through 3 months, broadly paralleling the analgesic trajectory. This pattern is consistent with the clinical view that better early control of ganglion-driven nociceptive activity may help improve sleep during the subacute period. The absence of a statistically significant between-group difference at 6 months should temper interpretation: the main added value of PRP + PRF in this cohort appeared to be acceleration of early recovery rather than a clearly demonstrated long-term superiority.

Lower pregabalin requirement at 3 months is also clinically meaningful, particularly in an older cohort with common comorbidities. Pregabalin is an established option for PHN and can improve both pain and pain-related sleep interference (17, 34). However, adverse effects such as dizziness, somnolence, edema, and cognition- or coordination-related symptoms are well recognized, and careful titration is especially important in older adults (15, 16). In our cohort, median pregabalin dose at 3 months was 0 mg/day in the PRP + PRF group and 150 mg/day in the PRF group. This finding may indicate less need for ongoing pharmacologic symptom suppression, but because tapering was not protocolized, it should be interpreted as a post-treatment medication-use outcome and possible mediator, not as an independent proof of efficacy.

The persistent-pain endpoint should be interpreted cautiously. At the 3-month post-treatment follow-up, the proportion of patients with NRS > 3 was numerically lower in the PRP + PRF group than in the PRF group, but the difference was not statistically significant. Because patients entered the study 30 to 90 days after rash onset, the post-rash interval at the 3-month post-treatment visit varied among patients. Although the visit occurred beyond the conventional 90-day threshold from rash onset in all included patients, this endpoint should not be equated with standardized PHN incidence. Our data therefore do not establish that PRP + PRF prevents PHN. The finding should be tested in a prospective design with a fixed post-rash PHN assessment time point.

Several limitations should be acknowledged. First, the retrospective single-center design and non-randomized treatment allocation introduce the possibility of selection bias and residual confounding. Although the primary result was robust to several sensitivity analyses, unmeasured factors such as patient preference, cost considerations, physician recommendation, detailed disease severity, prior antiviral treatment, and concomitant non-pregabalin medication use may have influenced the results. Second, the sample size was modest, particularly for the binary persistent-pain endpoint. Third, follow-up was limited to 6 months, and the between-group differences in NRS and PSQI were no longer statistically significant at that time. Fourth, although PRP was prepared using a standardized commercial workflow, PRP was not fully characterized; platelet concentration, leukocyte content, red blood cell contamination, activation status, and fold enrichment were not measured. Fifth, contrast was not routinely used before PRP administration, and therefore radiographic assessment of contrast spread was not available; procedural safety relied on anteroposterior/lateral fluoroscopic positioning, sensory and motor stimulation, and negative aspiration before injection. In addition, the exact number of treated thoracic segments was not available in the structured dataset. Finally, all included patients had thoracic segment involvement, limiting generalizability to cervical or lumbosacral zoster-related neuralgia.

## 5 Conclusion

Among patients with subacute thoracic herpes zoster neuralgia in this retrospective cohort, PRP combined with DRG-targeted PRF was associated with faster early improvement in pain and sleep and with lower post-treatment pregabalin requirement than PRF alone. The numerical reduction in persistent clinically meaningful zoster-related pain at 3 months was not statistically significant. These findings require confirmation in prospective multicenter studies with standardized treatment allocation, PRP characterization, concomitant-treatment protocols, and prespecified PHN assessment time points.

## Data Availability

The datasets generated and analyzed during the current study are not publicly available because they contain retrospective clinical data, but are available from the corresponding author on reasonable request and subject to institutional and ethical requirements.
